# Acute coronary syndrome with non-obstructive coronary arteries (ACS-NOCA) in patients with hypertrophic cardiomyopathy

**DOI:** 10.1186/s12872-021-02373-z

**Published:** 2021-11-19

**Authors:** Sarinya Puwanant, Angkawipa Trongtorsak, Chaisiri Wanlapakorn, Nattakorn Songsirisuk, Aekarach Ariyachaipanich, Smonporn Boonyaratavej

**Affiliations:** 1 Division of Cardiovascular Medicine, Department of Medicine, Faculty of Medicine, Chulalongkorn University, Rama IV Road, Bangkok, 10330 Thailand; 2 Cardiac Center, King Chulalongkorn Memorial Hospital, Thai Red Cross Society, Rama IV Road, Bangkok, 10330 Thailand

## Abstract

**Objectives:**

Our study aimed to determine the prevalence and prognosis of acute coronary syndrome with non-obstructive coronary artery (ACS-NOCA) in patients with hypertrophic cardiomyopathy (HCM).

**Methods and results:**

We enrolled a total of 200 consecutive patients with HCM over a 139-month period from 2002 to 2013. The study found that 28 patients (14% of overall patients, 51% of patients with ACS) had ACS-NOCA, and 18 patients (9% of overall patients, 86% of patients with acute MI) had MINOCA as initial clinical presentations. The highest prevalence of non-obstructive coronary artery disease (NOCA) in patients with HCM was found in acute ST-elevation myocardial infarction (STEMI) (100%), followed by non-STEMI (82%), and unstable angina (29%). Patients with ACS-NOCA had more frequent ventricular tachycardia and lower resting left ventricular (LV) outflow tract gradients than those with no ACS-NOCA (*p* < 0.05 for all). The ACS-NOCA group had a lower probability of HCM-related death compared with the no ACS-NOCA group and the significant coronary artery disease (CAD) group (p-log-rank = 0.0018).

**Conclusions:**

MINOCA or ACS-NOCA is not an uncommon initial presentation (prevalence rate 9–14%) in patients with HCM. NOCA was highly prevalent (51–86%) in patients with HCM presenting with ACS and had a favorable prognosis. Our findings highlight as a reminder that in an era of rapid reperfusion therapy, ACS in patients with HCM is not only a result of obstructive epicardial CAD, but also stems from the complex cellular mechanisms of myocardial necrosis.

## Introduction

Hypertrophic cardiomyopathy (HCM) is a common inherited disorder, with a prevalence of 0.2% in the general population [1]. Previous studies have reported that the prevalence of epicardial coronary artery disease (CAD) in HCM ranged from 10 to 53% [2–4]. Myocardial ischemia and/or infarction in the absence of epicardial obstructive CAD in patients with HCM have been described in several case reports [5–10]; however, little is known about the prevalence and prognosis of this entity. We, therefore, conducted this study to determine the prevalence and clinical significance of acute coronary syndrome with nonobstructive coronary artery (ACS-NOCA) in patients with HCM.

## Methods

### Study populations and definitions

The study protocol was approved by the Institutional Review Board of Faculty of Medicine, Chulalongkorn University. All methods were carried out in accordance with the tenets of the Declaration of Helsinki and the Chulalongkorn Research guidelines and regulations. We enrolled 200 consecutive patients with HCM attending a tertiary referral center between June 1, 2002 and December 31, 2013. The diagnosis of HCM was based on maximal left ventricular wall thickness ≥ 15 mm in one or more myocardial segments, or ≥ 13 mm with a family history of HCM in the absence of other conditions associated with ventricular hypertrophy. Myocardial wall thickness was assessed by two-dimensional transthoracic echocardiography and/or cardiac magnetic resonance imaging (MRI) by standard technique [11]. Acute coronary syndrome (ACS) was defined as a clinical syndrome of acute myocardial ischemia (unstable angina) or infarction (ST-segment elevation myocardial infarction or non-ST segment elevation myocardial infarction) [12]. Unstable angina (UA) was defined as clinical and electrocardiographic (ECG) findings of myocardial ischemia in the absence of acute cardiac myocyte necrosis or elevated biomarker level [13]. Acute myocardial infarction (MI) was defined as an acute myocardial injury with clinical evidence of acute myocardial ischemia and with detection of a rise and/or fall of cardiac enzyme markers and at least one of the following: angina or symptoms of myocardial ischemia, new ischemic ECG changes, or development of pathological Q waves [12]. Significant CAD was defined as ≥ 70% angiographically luminal stenosis in the major epicardial coronary branches, or ≥ 50% angiographically luminal stenosis in the left main coronary artery [14]. Nonsignificant CAD was defined as ≤ 50% luminal stenosis of epicardial coronary arteries [12]. ACS-NOCA was defined as a clinical syndrome of acute myocardial ischemia or MI with < 50% angiographically luminal stenosis in the major epicardial coronary branch. Myocardial infarction with nonobstructive coronary artery (MINOCA) was defined as a clinical syndrome that fulfilled the universal criteria for acute MI without obstructive coronary artery disease (≥ 50% diameter stenosis in a major epicardial vessel), and no overt cause for the clinical presentation at the time of angiography (e.g., classic features for Takotsubo cardiomyopathy) [15]. Echocardiogram, cardiac MRI, and coronary angiogram were analyzed by experienced observers who were blinded to the data of clinical outcomes.

### Patient and public involvement

Patients or the public were not involved in the study design, or conduct, or reporting, or dissemination plan of our research.

### Follow-up and outcomes

The follow-up period was defined as the time interval between the initial evaluation to the occurrence of death, or the date of the last contact. Death was determined by death certificates, telephone contacts, or electronic medical records. The follow-up data included overall death, HCM-related death, embolic stroke, and heart failure (HF), heart transplantation, and septal reductive therapy. HCM-related death included: sudden cardiac death (SCD), death associated with HF, or death due to embolic stroke. SCD was defined as unexpected death with or without documented ventricular tachycardia or ventricular fibrillation. HF was defined as a clinical syndrome characterized by the presence of symptoms and signs of congestion, or functional limitations, which may require hospital admission. Fatal embolic stroke was defined as death due to cardioembolic stroke, usually in the presence of atrial fibrillation.

### Statistical analysis

We calculated mean ± SD for continuous data and frequencies for categorical data. Chi-square tests were used for comparisons of categorical variables. Student’s t-test and Wilcoxon sum rank test were used for comparisons of continuous variables with normal and non-normal distributions, respectively. Survival curves were constructed according to the Kaplan–Meier method, and comparisons were performed using the log-rank test. All p-values were 2 sided and were considered significant when *p* < 0.05. Statistical analysis was performed with SPSS software, version 22.0 (Armonk, NY: IBM Corp) and JMP version 15.2 (SAS Institute Inc., Cary, North Carolina).

## Results

### Patients with HCM and ACS-NOCA

**Fig. 1:**
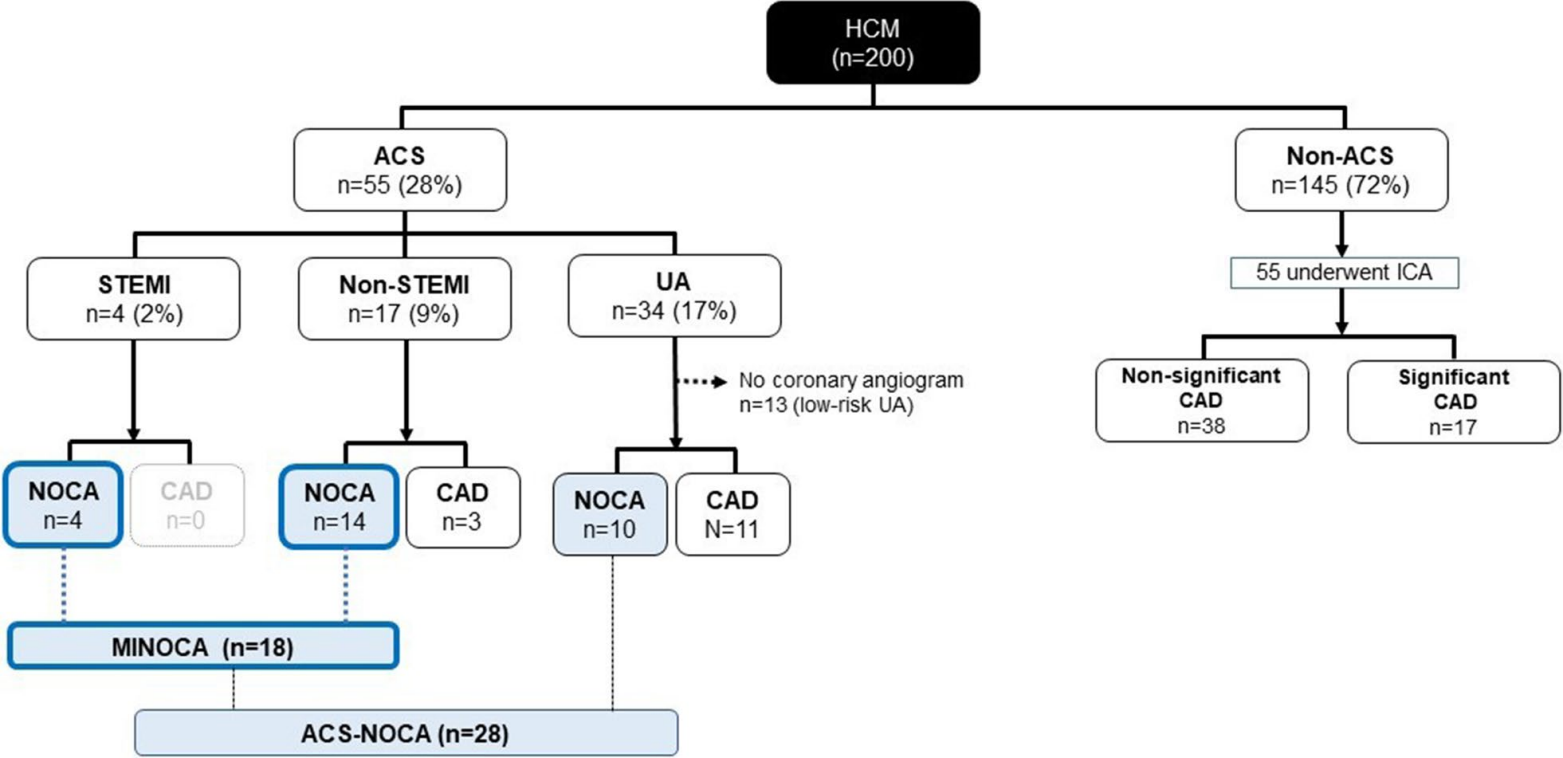
The proportions of acute coronary syndrome and its subtypes, non-obstructive coronary arteries, and coronary artery diseases in overall patients with hypertrophic cardiomyopathy

A total of 200 consecutive patients with HCM were included in the study. Mean age was 66 ± 16 years and 42% of patients were males. ACS as the initial clinical presentation was identified in 55 (28%) of 200 patients with HCM, 42 (23%) of whom underwent coronary angiogram. Of these, 28 patients had non-obstructive coronary arteries (NOCA) and the prevalence of ACS-NOCA in the study cohort was 14%. Of the 28 patients, 4 (14%) were STEMI, 14 (50%) were NSTEMI and 10 (36%) were UA. Figure 1 illustrate the proportions of ACS and its subtypes, NOCAs, and CADs in overall patients. Baseline clinical and echocardiographic characteristics of study patients are shown in Table 1. All patients with ACS had anginal symptoms at initial evaluation. Patients with ACS-NOCA more frequently had a history of ventricular tachycardia (VT) and lower resting left ventricular outflow tract (LVOT) gradients than those with no ACS-NOCA. Of 7 patients with ACS-NOCA who had a history of VT, 6 (86%) received implantable cardioverter defibrillator (ICD) treatment, and 7 (100%) and 1 (14%) were treated with beta-blocker and amiodarone, respectively. ICD implantation rates between patients with and without ACS-NOCA were not different. Of 14 patients with ACS and obstructive coronary arteries/CADs, 12 underwent percutaneous coronary revascularization with drug eluting stents, one underwent coronary bypass graft surgery, and one were medically treated due to a small obstructive lesion. Among the patient (50% were female) who underwent percutaneous coronary revascularization, 10 received dual antiplatelet (DAPT) for at least 12 months and 2 received monotherapy of P2Y12 inhibitor after 3 months of DAPT.

**Table 1:** Baseline demographic, clinical and echocardiographic characteristics of patients with HCM

	Overall (N = 200)	HCM with ACS-NOCA (n = 28)		HCM with no ACS-NOCA (n = 172)		*P* value^¶^	
Age (years)	66 ± 16	67 ± 14		66 ± 16		0.779	
Male [n, (%)]	83 (42%)	12 (43%)		71 (41%)		0.875	
Patients < 30 years [n, (%)]	5 (3%)	0		5 (3%)		0.361	
Age at diagnosis (years)	48 ± 17	47 ± 13		48 ± 17		0.354	
Family history of SCD [n, (%)]	16 (8%)	1 (4%)		15 (9%)		0.705	
Family history of HCM [n, (%)]	24 (12%)	4 (14%)		20 (12%)		0.688	
Syncope [n, (%)]	23 (12%)	2 (7%)		21(12%)		0.748	
History of VT [n, (%)]	27 (14%)	7 (25%)		20 (12%)		0.010*	
Diabetes [n, (%)]	49 (25%)	6 (21%)		43 (25%)		0.684	
Hypertension [n, (%)]	111 (56%)	14 (50%)		97 (56%)		0.527	
Atrial fibrillation [n, (%)]	15 (9%)	3 (11%)		12 (7%)		0.486	
Maximal Septal thickness (mm)	19 ± 6	18 ± 7		19 ± 6		0.467	
Resting LVOT gradient (mmHg) ^**‡**^	23 ± 30	15 ± 21		25 ± 31		0.044 *	
Reverse LV curvature [n, (%)]	69 (35%)	6 (21%)		63 (37%)		0.117	
LVEF < 50% [n, (%)]	11 (6%)	3 (11%)		8 (5%)		0.187	
Beta-blocker [n, (%)]	148 (74%)	21 (75%)		127 (74%)		0.896	
Calcium-channel blocker [n, (%)]	25 (13%)	2 (7%)		23 (13%)		0.355	
Amiodarone [n, (%)]	16 (8%)	1 (4%)		15 (9%)		0.705	
Warfarin [n, (%)]	47 (24%)	8 (29%)		39 (23%)		0.468	
ICD [n, (%)]	32 (16%)	6 (21%)		26 (15%)		0.392	
Surgical myectomy [n, (%)]	24 (12%)	0 (0%)		24 (13%)		0.021*	
Alcohol septal ablation [n, (%)]	5 (3%)	0		5 (3%)		0.328	
Heart Transplant [n, (%)]	4 (2%)	1 (6%)		3 (2%)		0.522	
Acute coronary syndrome							
STEMI [n, (%)]	4 (2%)	4 (22%)		0		< 0.001*	
NSTEMI [n, (%)]	17 (9%)	14 (50%)		3 (2%)			
Unstable angina [n, (%)]	34 (17%)	10 (36%)		24 (14%)			
Myocardial bridging [n, (%)]	4 (2%)	3 (11%)		1 (3%)		0.305	
Chronic coronary							
HCM related death [n, (%)]	42 (21%)	2 (7%)		40 (23%)		0.052	
NYHA						0.119	
Class I–II [n, (%)]	161 (81%)	26 (93%)		135 (79%)			
Class III–IV [n, (%)]	39 (9%)	2 (7%)		37 (21%)			

*ACS-NOCA* acute coronary syndrome with nonobstructive coronary artery, *ICD* implantable cardioverter defibrillator, *LV* left ventricular, *LVEF* left ventricular ejection fraction, *LVOT* left ventricle outflow tract, *NSTEMI* non ST-segment elevated myocardial infarction, *NYHA* New York Heart Association functional classification, *SCD* sudden cardiac death, *STEMI* ST-segment elevated myocardial infarction, *VT* ventricular tachycardia

^‡^Resting LVOT gradient was obtained and measured by using echocardiography

^¶^*P* value of the comparison between patients with HCM with and without ACS -NOCA

^*^Statistical significance

### Patients with HCM and MINOCA

Acute MI as the initial clinical presentation was identified in 21 (11%) of 200 patients with HCM; 18 patients had MINOCA (86% of patients with acute MI and 9% of overall patients with HCM). Of these, 4 (22%) were STEMI and 14 (78%) were NSTEMI, Fig. 1. Among 4 patients with STEMI, 3 had convex ST-segment elevation with biphasic T wave in V2-6 and one had concave upward ST-segment elevation in V2-6. Of 14 patients with NSTEMI, one had biphasic T-wave change, 4 had T-wave inversion, and 9 had ST-segment depression. The median troponin T in these patients was 4.56 (range: 0.1–372) ng/ml. Three patients (17%) with MINOCA had myocardial bridging on a coronary angiogram.

### Clinical outcomes

**Fig. 2:**
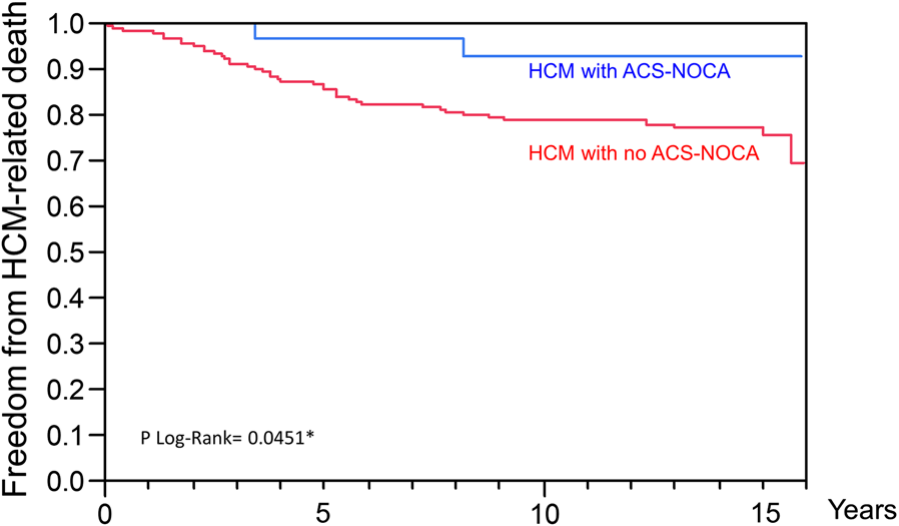
Survival Curves for Freedom from HCM-related Death between HCM with ACS-NOCA and HCM with no ACS-NOCA. ACS: acute coronary syndrome; NOCA: non-obstructive coronary arteries; HCM: hypertrophic cardiomyopathy

After a median follow-up time of 13 years, 51 deaths occurred including 9 deaths unrelated to HCM (e.g., cancer, infection); 42 (21%) of the deaths were HCM-related. Eight patients were lost to follow-up; all of them could not be contacted. There was a trend towards a low HCM-related death rate in patients with ACS-NOCA (2 [7%] in patients with ACS-NOCA vs. 40 [23%] in patients with no ACS-NOCA, *p* = 0.052). SCD secondary from VT was responsible for 7% of deaths in the ACS-NOCA group. The causes of death in patients with no ACS-NOCA included SCD (n = 8), HF (n = 30), and stroke (n = 2). Kaplan–Meier analysis indicated a trend of a lower rate of HCM-related death in the ACS-NOCA group compared with the no ACS-NOCA group (p log-rank = 0.0451; Fig. 2). Patients with ACS-NOCA had lower rates of myectomy compared with the no ACS-NOCA group (0% versus 13%; *p* = 0.021). Rates of heart transplantation and alcohol ablation were not different between the two groups (6% in the ACS-NOCA group versus 2% in the no ACS-NOCA group; *p* = 0.533 for heart transplant; and 0% in the ACS-NOCA group versus 3% in the no ACS-NOCA group; *p* = 0.328 for alcohol septal ablation).

**Fig. 3:**
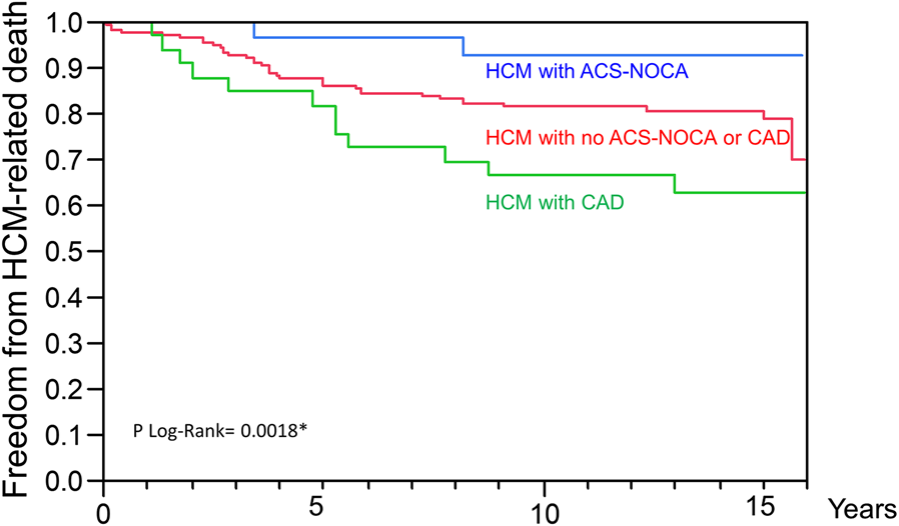
Survival Curves for Freedom from HCM-related Death among HCM with ACS-NOCA, HCM with no ACS-NOCA or CAD, and HCM with CAD. ACS: acute coronary syndrome; CAD: coronary artery disease; NOCA: non-obstructive coronary arteries; HCM: hypertrophic cardiomyopathy

Coronary angiography was performed in 97 (49%) of 200 patients, 31 of whom had significant CAD (17%). Of these, 14 patients presented with ACS, whereas the other 17 presented with either HF (n = 11), chronic stable angina (n = 5), or preoperative evaluation (n = 1). As shown in Fig. 3, the ACS-NOCA group had a lower probability of HCM-related death compared with the no ACS-NOCA group and the CAD group (p log-rank = 0.0018).

## Discussion

The major findings of the study were: (1) the prevalence of ACS-NOCA and MINOCA as an initial clinical presentation in patients with HCM was 14% and 9%, respectively; (2) the majority of patients (51%) with HCM presenting with ACS had non-obstructive coronary artery (NOCA); (3) patients with ACS-NOCA had a lower resting LVOT gradient and a lower rate of surgical myectomy; and (4) patients with HCM and ACS-NOCA had a favorable prognosis.

### Prevalence and characteristics of HCM patients with ACS-NOCA

We found that the prevalence of ACS-NOCA and MINOCA in the HCM cohort was 14% and 9%, respectively. Our findings underscore that ACS with non-obstructive epicardial coronary arteries in patients with HCM is not uncommon. Previous case reports described patients with MINOCA or ACS-MINOCA [5–10], however, there has been a paucity of case series data on the prevalence of this entity. Maron et al. [16] examined transmural MI in the absence of atherosclerosis in patients with HCM in cardiac autopsy and necropsy specimens. The prevalence rate of transmural MI with normal coronary artery in their study was 15% although those patients did not have angina at presentation. Yang et al. [17] reported that 21% of 91 patients with HCM and acute MI had nonstenotic coronary arteries. The prevalence rate of NOCA in our patients with acute MI and ACS was 86% and 51%, respectively. The explanation for a higher rate of NOCA in our cohort may include a higher rate of patients with acute NSTEMI undergoing coronary angiography. Yang et al. did not report the number of patients with acute MI (e.g., NSTEMI) who did not undergo coronary angiography in their study. Previous studies examining epicardial CAD in patients with HCM undergoing coronary angiography reported 47–80% rate of normal coronary arteries [3, 18]. However, these studies included HCM patients with no clinical context of ACS referred for coronary angiography. Walston et al. found that the classical ECG of acute MI was detected in 8 of the 33 (24%) HCM patients with normal coronary arteries [3], whereas classical ECG of acute MI in our study was found in 64% of patients with HCM and ACS-NOCA. In the study by Gupta et al. [19] comparing outcomes of acute MI in patients with HCM and patients with no HCM, NSTEMI was the most common type of MI accounted for 77% of patients with HCM. Similarly, NSTEMI accounted for the majority of ACS in our cohort. Pathophysiological mechanisms of MI in the absence of epicardial disease in patients with HCM were microvascular dysfunction, myocardial bridging, vasospasm, mismatch of myocardial oxygen demand and supply due to the hypertrophied muscle, and structure of intramural small vessels [20–22]. Microvascular dysfunction has been described in patients with HCM with angina [23, 24]. Foa et al. reported key findings of microvascular remodeling from myectomy specimens, which included luminal narrowing and thickening of the vascular wall [25]. Previous studies reported that the prevalence of myocardial bridging in patients with HCM diagnosed by coronary angiogram was 15–28% and by CT scan was 50–62% [2, 26–28]. Notably, previous studies included patients with HCM presenting with chronic stable angina and HF undergoing coronary angiogram or CT scan. In contrast, the prevalence of myocardial bridging in our study was low (3%). We hypothesized that myocardial bridging may a play role in chronic stable angina rather than in ACS-NOCA or MINOCA. Another mechanism for the angina described in HCM patients was the LVOT obstruction [22]. Dawn et al. [29] demonstrated that LVOT obstruction was a significant predictor for developing chest pain. Nonetheless, our patients with ACS-NOCA had a lower resting LVOT gradient compared with the no ACS-NOCA group. LVOT obstruction is a dynamic phenomenon and usually occurs with exercise. The proportion of LVOT obstruction in our study may be underestimated because we did not perform a routine provocative test in the echocardiography laboratory in all patients. Additionally, we excluded patients with chronic stable angina, HF, or evidence of ischemia by non-invasive testing of the analysis. We found that LV wall thickness was not different between the ACS-NOCA and no ACS-NOCA group. These findings suggest that microvascular disease, intramural coronary artery course, and endothelial dysfunction may play an important role for myocardial ischemia and/or infarction rather than LV mass or LV wall thickness. We found that patients with ACS-NOCA more frequently had a history of VT than those with no ACS-NOCA. Kwon et al. [30] and Suk et al. [30, 31] reported that the degree of myocardial scar or fibrosis assessed by cardiac MRI was strongly associated with a history of VT in patients with HCM. Although we did not examine the presence and extent of myocardial scar in this study, we hypothesized that patients with ACS-NOCA may have higher myocardial necrosis frequency or burden than those with no ACS-NOCA.

### Outcomes of HCM patients with ACS-NOCA

We demonstrated that patients with HCM and ACS-NOCA had a lower probability of HCM-related death compared to the no ACS-NOCA group. Similarly, Gupta et al. [19] demonstrated that patients with HCM with STEMI had lower in-hospital mortality and were less likely to receive revascularization compared with the non-HCM group. They reported that in-hospital mortality rates in HCM with STEMI and NSTEMI were 11% and 5%, respectively. Yang et al. [17] followed 91 patients with HCM and acute MI for 4.9 years, and they observed that the annual mortality rates were 6%. Notably, 21% of those patients had normal coronary arteries. We found that the HCM-related death rate in patients with HCM and ACS-NOCA at the median follow-up of 13 years was 7%. Compared with the mortality rate of ACS in the general population, patients with HCM and ACS-NOCA have a more favorable prognosis. Khan et al. found that the mortality of acute MI was 29.4%. [32] SNAPSHOT ACS study reported that mortality rates over 18 months of follow-up were 16.2% in STEMI, 16.3% in NSTEMI, and 6.8% in UA. [33] Additionally, Andrew et al. revealed that the mortality rate of 4,624 survivors after ACS was 6.8% [34].

We demonstrated that patients with HCM and significant epicardial CAD had the poorest prognosis while patients with ACS-NOCA had the most favorable prognosis. Sorujja et al. [18] demonstrated that, among patients with HCM undergoing coronary angiography, patients with HCM and severe or mild to moderate CAD had a higher cardiac death rate compared with patients with no CAD (adjusted *p* = 0.004). The 10-year survival rate for the endpoint of cardiac death in their study was 62%. Lazzeroni et al. [4] also demonstrated that mortality in patients with concomitant CAD and HCM was higher than HCM patients with no significant CAD. These data suggest that the epicardial coronary artery was the major determinant of prognosis in these patients. As the patients with significant CAD (n = 31) were included in the no ACS-NOCA group (n = 172). This may explain a poorer outcome in these patients. Although patients with ACS-NOCA more frequently had a history of VT, ICD implantations were performed in 86% of these patients. The proportion of ICD implantation rates, a family history of sudden cardiac death, age at the time of HCM diagnosis, diabetes, or other comorbidities were similar in both groups. Among patients with ACS with significant CAD as initial presentation, 16% of these received monotherapy of P2Y12 inhibitor after 3 months of DAPT therapy following percutaneous revascularization. A recent meta-analysis demonstrated that P2Y12 inhibitor monotherapy was associated with a similar risk of adverse cardiovascular outcomes, especially in women [35].

### Study limitations

The primary limitation of the present study is the small sample size. Nevertheless, this is the first study to highlight the prognosis of patients with HCM and MINOCA or ACS-NOCA. As ACS was a clinical syndrome, we did not include data of stress imaging to document scar/infarction or ischemia. Furthermore, the study excluded patients with SCD or VT from ACS, and the study cohort was conducted in a tertiary referral center where the majority of patients were referred for septal reductive surgery or advanced care of HCM. This could limit the applicability and generalizability of our findings to HCM patients in the community cohort. Lastly, this study is limited by its retrospective nature.

## Conclusions

Our study demonstrated that MINOCA/ACS-NOCA is not an uncommon initial presentation (prevalence rate 9–14%) in patients with HCM. NOCA was highly prevalent (51–86%) in HCM patients with ACS who underwent coronary angiography. Our findings highlight as a reminder that in the era of rapid reperfusion therapy ACS in patients with HCM is not only a result of obstructive epicardial CAD, but also stems from the complex cellular mechanisms of myocardial necrosis. A careful review of previous ECG or performing a focused cardiac ultrasound (FOCUS) or an echocardiogram may be helpful in guiding the appropriate therapy. ACS-NOCA was not associated with an increased mortality.

## Data Availability

The datasets used and/or analyzed during the current study available from the corresponding author on reasonable request.
